# Your brain on speed: cognitive performance of a spatial working memory task is not affected by walking speed

**DOI:** 10.3389/fnhum.2014.00288

**Published:** 2014-05-08

**Authors:** Julia E. Kline, Katherine Poggensee, Daniel P. Ferris

**Affiliations:** ^1^Department of Biomedical Engineering, University of MichiganAnn Arbor, MI, USA; ^2^School of Kinesiology, University of MichiganAnn Arbor, MI, USA

**Keywords:** locomotion, EEG, brain imaging, dual-tasking, spatial working memory

## Abstract

When humans walk in everyday life, they typically perform a range of cognitive tasks while they are on the move. Past studies examining performance changes in dual cognitive-motor tasks during walking have produced a variety of results. These discrepancies may be related to the type of cognitive task chosen, differences in the walking speeds studied, or lack of controlling for walking speed. The goal of this study was to determine how young, healthy subjects performed a spatial working memory task over a range of walking speeds. We used high-density electroencephalography to determine if electrocortical activity mirrored changes in cognitive performance across speeds. Subjects stood (0.0 m/s) and walked (0.4, 0.8, 1.2, and 1.6 m/s) with and without performing a Brooks spatial working memory task. We hypothesized that performance of the spatial working memory task and the associated electrocortical activity would decrease significantly with walking speed. Across speeds, the spatial working memory task caused subjects to step more widely compared with walking without the task. This is typically a sign that humans are adapting their gait dynamics to increase gait stability. Several cortical areas exhibited power fluctuations time-locked to memory encoding during the cognitive task. In the somatosensory association cortex, alpha power increased prior to stimulus presentation and decreased during memory encoding. There were small significant reductions in theta power in the right superior parietal lobule and the posterior cingulate cortex around memory encoding. However, the subjects did not show a significant change in cognitive task performance or electrocortical activity with walking speed. These findings indicate that in young, healthy subjects walking speed does not affect performance of a spatial working memory task. These subjects can devote adequate cortical resources to spatial cognition when needed, regardless of walking speed.

## INTRODUCTION

In everyday life, people perform complex cognitive tasks while walking through various environments. This has led to a variety of studies on how humans dual-task cognitive and locomotor movements. However, the results from these studies are mixed. Recent reviews highlight the conflicting results in regard to cognitive and motor performance during dual-tasking (Al-Yahya et al., 2011; Schaefer and Schumacher, 2011; Kelly et al., 2012b). Elderly individuals (Lindenberger et al., 2000; Simoni et al., 2013; Venema et al., 2013) and individuals with neurological deficits (Sheridan et al., 2003; Camicioli et al., 2006; Panyakaew and Bhidayasiri, 2013) tend to exhibit increased gait variability and decreased mental performance when dual-tasking walking and a cognitive task. Data from young, healthy subjects do not follow such a clear pattern. Some studies have shown cognitive-motor dual task cost in young, healthy adults (Grabiner and Troy, 2005; Al-Yahya et al., 2009; Verrel et al., 2009; Szturm et al., 2013), but other studies have found that young, healthy subjects either have no dual-task effect (Grubaugh and Rhea, 2014) or a reduced magnitude effect (Melzer and Oddsson, 2004; Siu et al., 2008; Srygley et al., 2009; Yogev-Seligmann et al., 2010). Furthermore, the observed dual-task effect on kinematic or kinetic variables often differs across studies.

Some of the discrepancies in dual-tasking results across studies may be related to the type of cognitive task chosen. The cognitive tasks that have been used for gait dual-tasking studies include an N-back task (Verrel et al., 2009), Stroop tasks (Melzer and Oddsson, 2004; Grabiner and Troy, 2005; Siu et al., 2008; Kelly et al., 2012a), a serial subtraction and a phoneme-monitoring task (Al-Yahya et al., 2009; Srygley et al., 2009), an N-back task and a spatial attention task (Nadkarni et al., 2010), a verbal fluency task (Yogev-Seligmann et al., 2010), and an automated operation span task (Grubaugh and Rhea, 2014). All of these tasks except the automated operation span task (Grubaugh and Rhea, 2014) had some effect on gait parameters. The N-back, Stroop, serial subtraction, verbal fluency, and automatic operation span tasks are all non-spatial working memory tasks. If walking is similar to upper limb motor tasks, it will engage cortical areas that are also involved in spatial working memory.

Work by Anguera et al. (2010) supports the idea that spatial working memory tasks overlap with sensorimotor brain areas. Their subjects performed a visuomotor adaptation task that involved manipulating a joystick to hit a visual target, and they also separately performed a spatial working memory task that involved mental rotation. Activation in the dorsolateral prefrontal cortex and the bilateral inferior parietal lobule overlapped between the motor and mental tasks. Moreover, spatial working memory performance predicted the rate of visuomotor adaptation. This suggests that upper limb visuomotor adaptation and spatial working memory likely share mental resources (Anguera et al., 2010).

Another factor that may result in discrepancies in dual-task findings across studies is walking speed. Walking speed fundamentally changes the dynamics of human gait. At faster speeds there is less time to make changes in limb movement for each step, and the body’s inertia is greater compared with slower speeds. These biomechanical changes are related to an increase in mechanical energy and passive dynamics at faster walking speeds compared with slower walking speeds (McGeer, 1993; Collins et al., 2005; Kuo, 2007). In addition, evidence from individuals with spinal cord injuries suggests that faster walking speeds rely more on spinal reflex pathways and spinal neural networks compared with slower walking speeds (Maegele et al., 2002; Beres-Jones and Harkema, 2004; Ferris et al., 2004; Behrman et al., 2005). Results from functional near-infrared spectroscopy indicate that humans have increased frontal brain activity at faster walking speeds compared with slower walking speeds (Harada et al., 2009). All of these data suggest that walking speed is likely to have an effect on brain activity during walking. By studying our subjects across a range of speeds, we could examine speed dependent differences in how the spatial working memory task affected brain activation and behavioral performance measures.

The purpose of this study was twofold. First, we wanted to determine if concurrent performance of a spatial working memory task affects human walking dynamics in young, healthy subjects across a range of walking speeds. Second, we wanted to determine if walking speed affects cognitive task performance and electrocortical activity during a working memory task across those same walking speeds. Reported differences between young and older subjects and across studies might be due to differences in the specific walking speeds studied or the fact that there was no control of walking speed across the cognitive task and no cognitive task conditions. We studied young, healthy subjects because they generally use a wide range of walking speeds in everyday life, and they are more cognitively capable compared with older subjects. We chose a Brooks spatial working memory task, because a balance task during standing disrupted performance on the spatial but not the non-spatial Brooks task (Kerr et al., 1985), and because there seems to be an overlap in spatial working memory brain regions and sensorimotor brain regions (Anguera et al., 2010).

Specifically, we had two hypotheses about cognitive and motor dynamics. We hypothesized that walking kinematics would show signs of stability challenges when subjects performed the spatial working memory task compared with when they had no cognitive task. Increases in gait variability or wider step widths are both signs that humans are adapting to stability challenges. We also hypothesized that walking at higher speeds would decrease the performance of the spatial working memory task and decrease the related electrocortical activity. If faster walking speeds require greater cortical attention for control, then cognitive performance and the related electrocortical activity should both decrease with walking speed. In this study, we measured event-related spectral power synchronized to the presentation of a stimulus as our metric of electrocortical activity across conditions. To address these questions, our subjects stood (0.0 m/s) and walked at a range of speeds (0.4–1.6 m/s) on a treadmill, with and without performing the Brooks spatial working memory task.

## MATERIALS AND METHODS

### DATA COLLECTION

Twenty healthy volunteers completed this study (18 males and 2 females, age range 18–39). All study procedures were approved by the University of Michigan Human Subjects Internal Review Board. All subjects provided written informed consent before participating.

**FIGURE 1:**
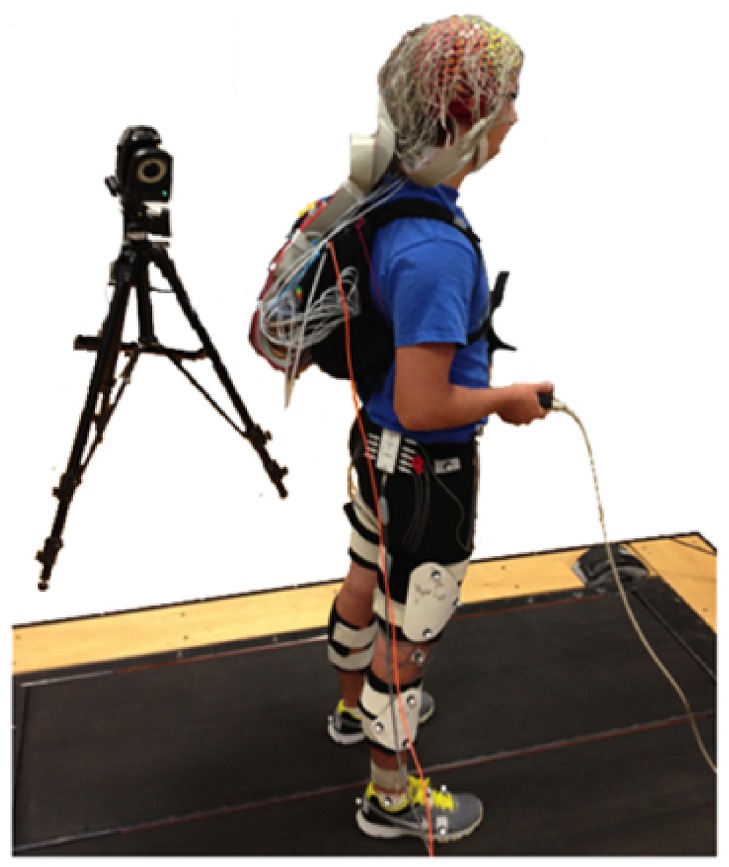
**20 subjects stood (0.0 m/s) and walked (0.4, 0.8, 1.2, and 1.6 m/s) on a treadmill while continuously performing the Brooks spatial working memory task.** We recorded EEG, kinematics, ground reaction forces, and response data from the Brooks task.

Subjects stood (0.0 m/s) and walked (0.4, 0.8, 1.2, and 1.6 m/s) on a treadmill with and without performing the Brooks spatial working memory task (Brooks, 1967). We recorded electroencephalography (EEG), motion capture data, ground reaction forces, and response data from the Brooks task (**Figure 1**). We fit each subject with an appropriately sized EEG cap. Before recording EEG data, we marked the position of each electrode on the subject’s head using a Zebris digitizer (Zebris, Germany). We filled the wells of the EEG cap with conductive gel and plugged the EEG electrodes into the holes. We ensured that all electrode offsets were <20 mV. We collected the EEG data at 512 Hz with a 264-channel active electrode array (ActiveTwo amplifier Biosemi, Amsterdam, Netherlands). The EEG signals were initially referenced to a common reference. We placed reflective markers on the subjects’ calcanei and recorded motion capture marker data at 100 Hz using 10 Vicon motion capture cameras (Vicon, Los Angeles, CA, USA) placed around the perimeter of the room. We created an automated version of the Brooks task using the Simulink toolbox (The MathWorks, Inc.) and collected response data in real-time using the dSPACE real-time interface (RTI) (dSPACE INC, Wixom, MI, USA).

### BROOKS TASK

**FIGURE 2:**
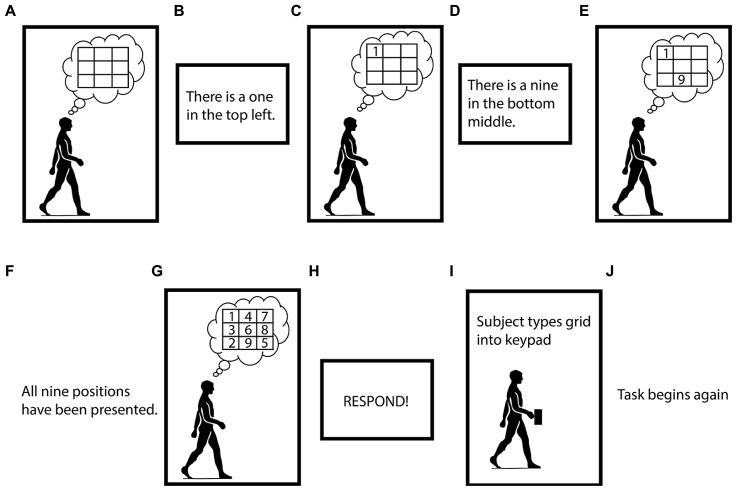
**Visual depiction of the Brooks spatial working memory task.** Panels **(A–J)** indicate the order of the task. Subjects performed the Brooks task continuously for 5 min intervals.

The Brooks spatial working memory task uses visuospatial working memory (Brooks, 1967). In our version of the task, we asked the subject to imagine an empty 3-by-3 grid. Then, a screen one meter in front of the subject instructed him or her to visualize the digits one through nine in randomized positions in the grid. Digits and their associated positions were presented, one at a time. Each stimulus (a digit and its position) appeared on the screen and remained there for 2 s, after which it vanished, and there was a 2 s pause before the next stimulus appeared. The subject had to maintain all the digits and their positions in working memory to successfully fill in the entire grid. It took 32 s for the subject to get all the information they needed to complete one grid. After all nine stimuli were presented, there was a 4 s pause, after which the screen prompted the subject to type the imagined grid, row-wise, into their hand-held keypad. Once the subject had pressed nine keys on their keypad the Brooks task began again (**Figure 2**). Subjects were not permitted to change any digits of their response once they had been entered. Subjects typically completed five or six runs of the Brooks task during each 5-min trial.

Subjects performed the Brooks spatial working memory task continuously for 5-min intervals seven total times. Subjects began by performing the task while standing. Next, they alternated between walking with the cognitive task and walking without the cognitive task for four trials. Then, they performed a second cognitive task trial while standing. Next, they again alternated between walking with the cognitive task and walking without the cognitive task for four trials, and finally, they performed the cognitive task while standing for a third time. The order of the speeds for the walking trials was randomized across subjects to avoid order effects, but all subjects walked at all four speeds with and without the cognitive task. We instructed the subjects to respond to the cognitive task as quickly and accurately as possible, and we gave them no instructions about how to walk.

### BROOKS TASK ANALYSIS

We gave the subjects two scores for each Brooks task trial, a percent correct score and a reaction time score. Percent correct was the number of digits placed correctly divided by the total number of presented digits. Reaction time was the average time that elapsed between the respond prompt and the ninth keystroke of the subject’s response. We compared the results across walking speeds using a one-way repeated measures analysis of variance (ANOVA) and the Bonferroni correction *post hoc* test in SPSS version 21 (SPSS IBM, Armonk, NY, USA).

### GAIT KINEMATICS ANALYSIS

To analyze the data with respect to the gait cycle, we used custom scripts in Visual3D (C-Motion, Germantown, MD, USA) to identify gait events from the calcaneus marker motion capture data. Specifically, we identified heel strikes times by finding the minimum values for the position of the calcaneus marker in the z direction. The length for each step was the absolute value of the distance in the y direction between the right and left calcaneus markers at heel strike. The width for each step was the absolute value of the distance in the x direction between the right and left calcaneus markers at heel strike. We calculated the standard deviation of the step length and step width values to obtain step length variability and step width variability values. All values were divided by each subject’s leg length to create unitless measures. We performed a two-way repeated measure ANOVA with speed and task (Brooks task vs. no task) as factor levels for step length, step length variability, step width, and step width variability. We set the significance level at *p* < 0.05 with a Bonferroni correction *post hoc* test.

We also performed a more finely grained analysis of step variability between the encoding and retrieval periods of the Brooks task. We broke up the biomechanical data into periods of encoding, which is the time period between the start of a task trial (mental grid is empty) and the respond prompt (grid is filled), and retrieval, which is the time period between the respond prompt and the ninth keystroke of the subject’s response. We calculated step length, step width, step length variability, and step width variability as above. We performed statistical analyses as above, with speed and task period (encoding period vs. retrieval period) as factor levels.

### EEG DATA ANALYSIS

We post-processed the EEG data using custom scripts in the open-source MATLAB toolbox, EEGLAB (Delorme and Makeig, 2004). We merged all EEG recordings from a single subject into one dataset and high-pass filtered the data above 1 Hz. We removed channels exhibiting substantial artifact on the basis of: (1) magnitude, with channels exhibiting values <30 or >10000 μV removed, (2) kurtosis, with channels >3 standard deviations from the mean removed, (3) correlation, with channels measuring voltages that are uncorrelated (*r* ≤ 0.4) with the surrounding channel voltages for more than 0.01% of the time removed, and (4) standard deviation, with channels measuring voltages that are substantial more variability relative to other channel voltages as measured by standard deviation removed. These cutoffs were based on the work of Gwin et al. (2010). We identified and rejected noisy frames, or time periods of EEG data exhibiting high power across all channels (greater than 1.6 times the interquartile range of the channels). For some subjects, we made minor adjustments to these values to ensure that all the noisy channels and frames were removed. On average, we rejected 130.5 channels (range, 108–153; std. dev., 14.0). We re-referenced the remaining channels to an average reference.

To the cleaned data sets, we applied adaptive mixture independent component analysis (AMICA; Palmer et al., 2006, 2008) to transform the EEG channel data into temporally independent component signals (ICs; Makeig et al., 1996). We used the DIPFIT function in EEGLAB (Oostenveld and Oostendorp, 2002) to model each IC as an equivalent current dipole within a boundary element head model based on the MNI (Montreal Neurological Institute) brain. We removed ICs from further analysis if the best-fit dipole accounted for less than 85% of the scalp map variance (Gwin et al., 2011), or if the scalp map or spectra were indicative of an eye or muscle artifact (Jung et al., 2000a,b).

We clustered ICs across all 20 subjects based on similarities in dipole location, scalp topography, and spectra using a *k*-means clustering algorithm that is available in EEGLAB. We made 20 clusters and retained clusters containing ICs from more than half of the subjects (>10) for further analysis.

For each cluster, we created an event-locked plot of spectral power change around each stimulus during the Brooks spatial working memory task, defined as the presentation of a digit and its position. We computed the power spectrum for each IC for every stimulus. We averaged the power spectrum over all stimuli for each IC and over all ICs for each cluster. To allow spectral changes over time to be easily visualized, we subtracted a baseline, which was the average spectrum over all time points, from the spectrum at each time point. These plots, showing spectral change from baseline, are referred to as event-related spectral perturbations (ERSPs; Makeig, 1993; Gwin et al., 2011). For the ERSP plots, time zero is stimulus onset. We used bootstrapping methods available in EEGLAB (Delorme and Makeig, 2004) to determine regions of significant difference from baseline (*p* < 0.05).

We wanted to determine if the ERSP data from the cognitive task showed a significant trend across the five walking speeds (0.0–1.6 m/s). Each ERSP plot is made up of 507 frequency bins and 200 time bins, yielding a total of 101,400 TxF bins. For each TxF bin, we used the MATLAB nlmefit function to fit a group level slope that best represented the change in raw spectral power across walking speed (from 0.0 to 1.6 m/s) for all independent components in the cluster. The nlmefit function fits a model where each model parameter is the sum of a fixed and random effect (mixed effect). For our model, walking speed was a mixed effect. After fitting the model at each TxF bin, we computed a *p*-value representing the significance of the slope at that bin. Finally, we accounted for family wise error rate by subjecting all 101,400 *p*-values for each cluster to a false discovery rate algorithm (http://go.warwick.ac.uk/tenichols/software/fdr/FDR.m). False discovery rate (Genovese et al., 2002) controls the expected proportion of false positives. We set the accepted false discovery rate to 5%. Based on the assumption of positive dependence among observations, the false discovery rate algorithm generated a new *p*-value for significance.

## RESULTS

### COGNITIVE TASK RESULTS

Subjects performed the Brooks spatial working memory task equally well at all walking speeds. Responses had an average percent correct around 50% regardless of walking speed (**Table 1**). Because each position had to be filled with one of nine digits, 11% accuracy reflects chance level performance. Subjects filled in all nine numbers in the grid after about 11–12 s, regardless of walking speed (**Table 1**). Statistically, there was no significant difference by speed for either parameter (**Table 1**).

**Table 1:** Average percent correct and average reaction time for the Brooks spatial memory task at all speeds.

Speed	Average percent correct	Average reaction time (s)
Stand (0.0 m/s)	48.3 (12.8)	11.1 (3.5)
0.4 m/s	51.3 (14.7)	11.5 (4.1)
0.8 m/s	49.8 (18.2)	11.9 (4.3)
1.2 m/s	53.3 (12.8)	11.8 (4.9)
1.6 m/s	46.0 (16.4)	10.9 (4.1)
*p*-value	0.18	0.44
*F*-value	1.798	1.000
df	4	4
Error (df)	76	76

*Data are the mean with the standard deviation in parentheses. The values for the Brooks task during standing is the average of the three standing Brooks task trials. The bottom two rows give the ANOVA results (*F* and *p*-values) for percent correct and reaction time across speeds for the Brooks task. There were no significant changes with speed in these task parameters.*

### GAIT KINEMATICS RESULTS

#### Brooks task vs. walking alone

**FIGURE 3:**
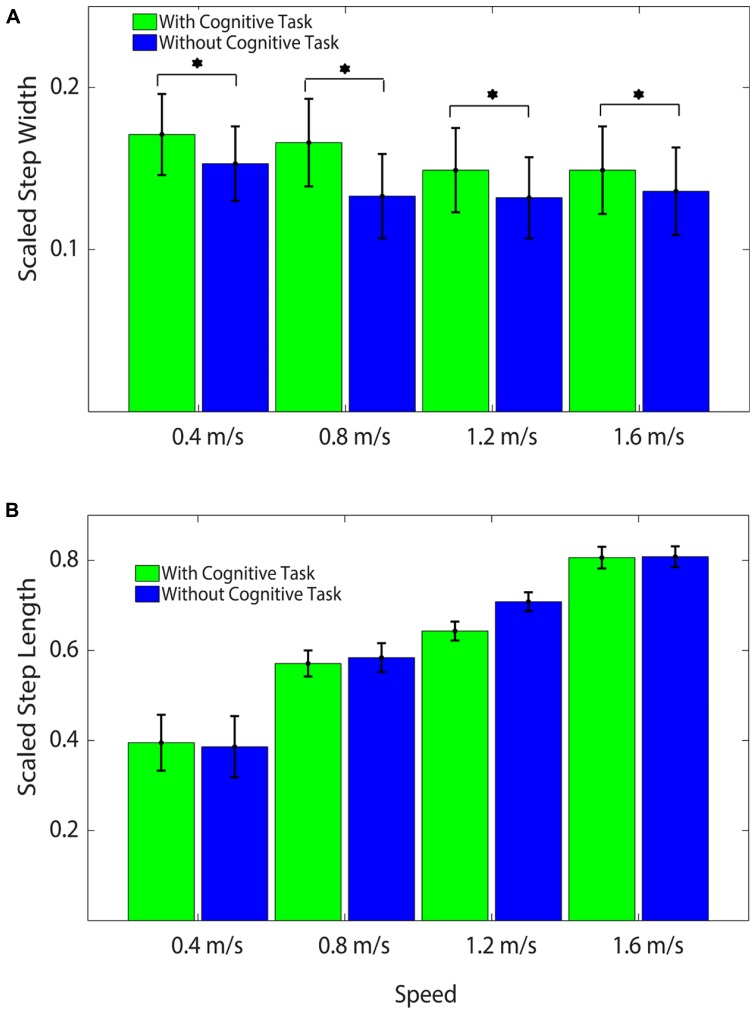
**(A)** Average Scaled Step Widths at all speeds with and without the cognitive task. Subjects stepped significantly wider with the cognitive task than without the cognitive task at all speeds. Asterisks indicate significant differences between conditions. All values were scaled by each subject’s leg length to create unitless measures. **(B)** Average Scaled Step Lengths at all speeds with and without the cognitive task. There were no significant differences by task across speed. All values were scaled by each subject’s leg length to create unitless measures.

The addition of the Brooks spatial working memory task had some limited effects on walking kinematics. At all walking speeds, step width increased significantly with the addition of the Brooks task compared with walking without the Brooks task (ANOVA, *F* = 22.62, *p* < 0.001; Bonferroni, *p* < 0.05; **Figure 3A**, **Table 2**). There were no significant differences between cognitive task and no cognitive task for step length (ANOVA, *F* = 1.74, *p* = 0.20; **Figure 3B**). Step length variability and step width variability also were not significantly different between cognitive task and no cognitive task across speeds (ANOVA, *F* = 1.85, *p* = 0.051 and *F* = 1.16, *p* = 0.30, respectively; **Table 2**). There was no interaction between speed and task condition for each of the four outcomes: step length, step length variability, step width, or step width variability (**Table 2**).

**Table 2:** ANOVA table showing *F* and *p*-values for speed, task, and speed × task interaction.

	Speed (df = 3)		Task (df = 1)		Speed × task		*Post hoc* test	
	*F*-value	*p*-value	*F*-value	*p*-value	*F*-value	*p*-value	By speed *p* <0.05	By task *p* <0.05
Step length	499.08	<0.001	1.74	0.20	0.90	0.46	All but 0.8 from 1.2	–
Step width	6.93	0.003	22.62	<0.001	0.53	0.67	0.4 from 1.2, 1.6	ALL
Step length variability	3.18	0.051	1.85	0.19	0.81	0.50	–	–
Step width variability	2.65	0.08	1.16	0.30	0.39	0.76	–	–

*By speed, all but one crosswise comparison (0.8 and 1.2 m/s) of step length values were significantly different from each other (ANOVA, *F* = 499.08, *p* < 0.001; Bonferroni, *p* < 0.05), and step width values were significantly different between 0.4 and 1.2 and 0.4 and 1.6 m/s (ANOVA, *F* = 6.93, *p* = 0.003; Bonferroni, *p* < 0.05). By task, step width was significantly greater with the cognitive task than without the cognitive task at all speeds (ANOVA, *F* = 22.62, *p* < 0.001; Bonferroni, *p* < 0.05).
*

Some gait kinematic parameters varied with walking speed, as expected. When comparing the different speeds with each other, step length increased with speed (ANOVA, *F* = 499.08, *p* < 0.001; Bonferroni, *p* < 0.05). Step width decreased at higher speeds (ANOVA, *F* = 6.93, *p* = 0.003; Bonferroni, *p* < 0.05). There were no significant differences in step length variability or step width variability between speeds (**Table 2**).

#### Encoding period vs. retrieval period for Brooks task

Walking kinematics during the Brooks task differed based on task period. Step length decreased during retrieval compared with encoding (ANOVA, *F* = 6.53, *p* = 0.02; **Table 3**). There was also a significant interaction effect between speed and task period on step length (ANOVA, *F* = 6.63, *p* = 0.004; **Table 3**). Both step width and step width variability increased during retrieval (ANOVA, *F* = 35.41, *p* < 0.001 and *F* = 16.33, *p* = 0.001, respectively; **Table 3**) compared with encoding. Step length variability did not show a statistically significant difference between encoding and retrieval (ANOVA, *F* = 3.07, *p* = 0.097; **Table 3**).

**Table 3:** ANOVA table showing *F* and *p*-values for speed, task period, and speed × task period interaction.

	Speed (df = 3)		Task period (df = 1)		Speed × task period		*Post hoc* test	
	*F*-value	*p*-value	*F*-value	*p*-value	*F*-value	*p*-value	By speed *p* <0.05	By task period *p* <0.05
Step length	988.22	<0.001	6.53	0.02	6.63	0.004	ALL	ALL
Step width	3.49	0.04	35.41	<0.001	2.42	0.10	0.4 from 1.2	ALL
Step length variability	4.68	0.016	3.07	0.097	0.64	0.60	0.4 from 1.2	–
Step width variability	1.52	0.25	16.33	0.001	0.77	0.53	–	ALL

*Step length showed a statistically significant interaction between speed and task period (ANOVA, *F* = 6.63, *p* = 0.004) as well as an increase in step length at higher speeds and during encoding (ANOVA, *F* = 988.22, *p* < 0.001; Bonferroni, *p* < 0.05 and *F* = 6.53, *p* = 0.02, respectively). Step width and step width variability increased during retrieval (ANOVA, *F* = 35.41, *p* < 0.001 and *F* = 16.33, *p* = 0.001, respectively). Step width and step length variability were significantly lower at 1.2 m/s than at 0.4 m/s (ANOVA, *F* = 3.49, *p* = 0.04 and *F* = 4.68, *p* = 0.016, respectively; Bonferroni, *p* < 0.05).*

Gait kinematic parameters again varied with walking speed. For steps taken in the encoding and retrieval periods, there was a significant increase in step length with speed (ANOVA, *F* = 988.22, *p* < 0.001; Bonferroni, *p* < 0.05; **Table 3**). Both step width and step length variability were significantly lower at 1.2 m/s than at 0.4 m/s (ANOVA, *F* = 3.49, *p* = 0.04 and *F* = 4.68, *p* = 0.016, respectively; Bonferroni, *p* < 0.05; **Table 3**). There were no other statistically significant differences in step width, step length variability, or step width variability at any other speeds.

### EEG RESULTS

Twelve independent component clusters met our criteria for further analysis (>10 subjects). Of the twelve clusters, six showed significant spectral power shifts temporally linked to stimulus presentation (**Table 4**).

**Table 4:** Centroid location for all clusters of electrocortical sources containing ICs from more than ten subjects that showed significant spectral power shifts temporally linked to stimulus presentation.

Functional area	Brodmann area	Subjects (#)	ICs (#)
Left somatosensory association cortex	7	13	33
Central somatosensory association cortex	7	12	35
Right somatosensory association cortex	7	11	17
Central posterior cingulate cortex	31	12	35
Right superior parietal lobule	5	13	39
Central premotor and supplementary motor area	6	12	26

*Brodmann area (BA) is listed as well as the number of independent components (ICs) in each cluster.
*

**FIGURE 4:**
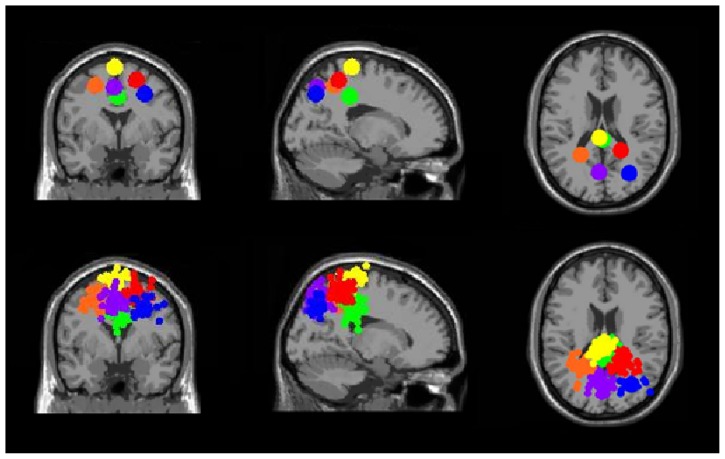
**Clusters of electrocortical sources with significant spectral shifts time-locked to stimulus presentation during the Brooks spatial working memory task.** Green is the central posterior cingulate (BA 6), blue is the right somatosensory association cortex (BA 7), orange is the left somatosensory association cortex (BA 7), purple is the middle somatosensory association cortex (BA 7), red is the right superior parietal lobule (BA 5), and yellow is the central premotor and supplementary motor cortex (BA 6). From left to right, the top three images show the centroid locations for each cluster from a horizontal, coronal, and sagittal view. The bottom three images show the independent component dipoles for each cluster from the same three perspectives.

**FIGURE 5:**
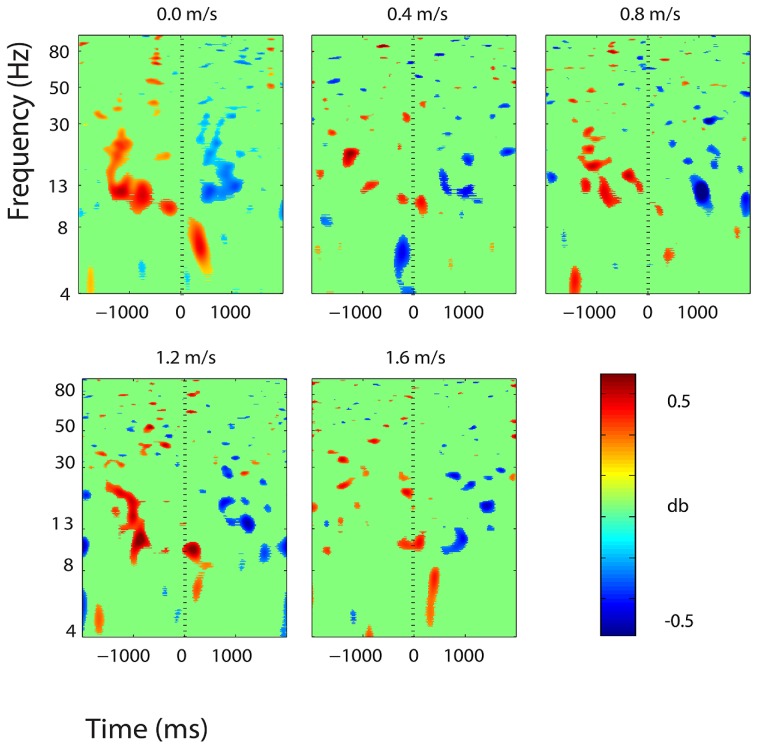
**Event-related spectral perturbation (ERSP) plots showing power change around the presentation of a stimulus (a digit and its position in an imagined grid) in the left somatosensory association cortex (BA 7).** Red represents a power increase from baseline and blue represents a power decrease from baseline. We set non-significant differences to 0 dB (green). The stand condition ERSP is the average of all three standing Brooks spatial working memory task trials.

**FIGURE 6:**
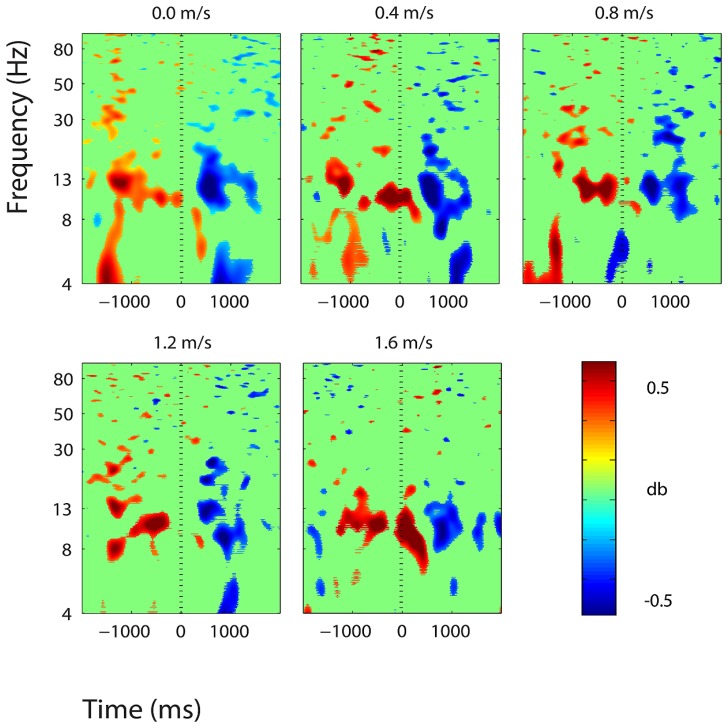
**Event-related spectral perturbation plots showing power change around the presentation of a stimulus (a digit and its position in an imagined grid) in the central somatosensory association cortex (BA 7).** Red represents a power increase from baseline and blue represents a power decrease from baseline. We set non-significant differences to 0 dB (green). The stand condition ERSP is the average of all three standing Brooks spatial working memory task trials.

**FIGURE 7:**
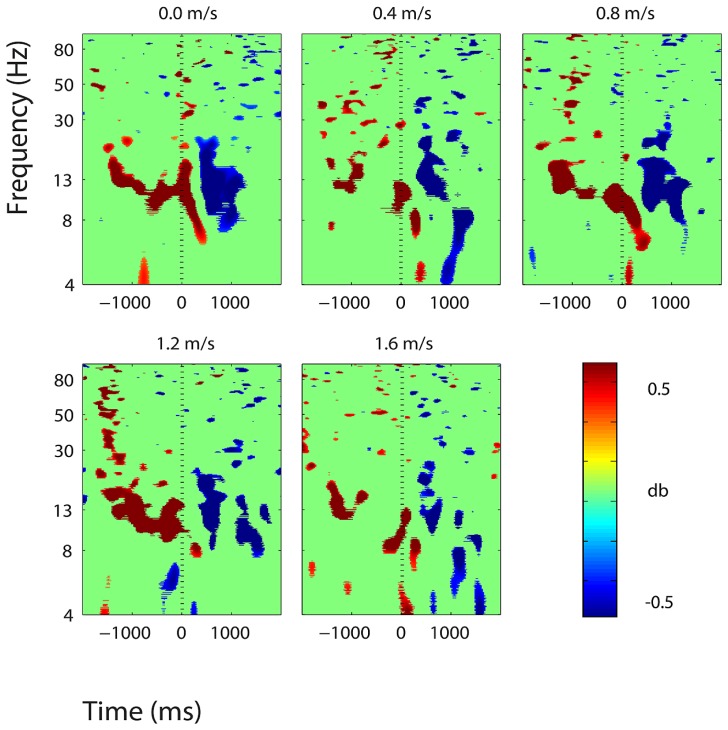
**Event-related spectral perturbation plots showing power change around the presentation of a stimulus (a digit and its position in an imagined grid) in the right somatosensory association cortex (BA 7).** Red represents a power increase from baseline and blue represents a power decrease from baseline. We set non-significant differences to 0 dB (green). The stand condition ERSP is the average of all three standing Brooks spatial working memory task trials.

Three clusters in the somatosensory association cortex had large changes in spectral power that were temporally linked to stimulus presentation and were consistent for all walking speeds. These cluster centroids were located in left, central, and right somatosensory association cortex (**Figure 4**). In all three somatosensory association clusters, alpha (8–13 Hz) power increased prior to stimulus presentation and decreased following stimulus presentation (**Figures 5**–**7**). The increase in spectral power likely represented an anticipatory effect given that it preceded stimulus presentation.

**FIGURE 8:**
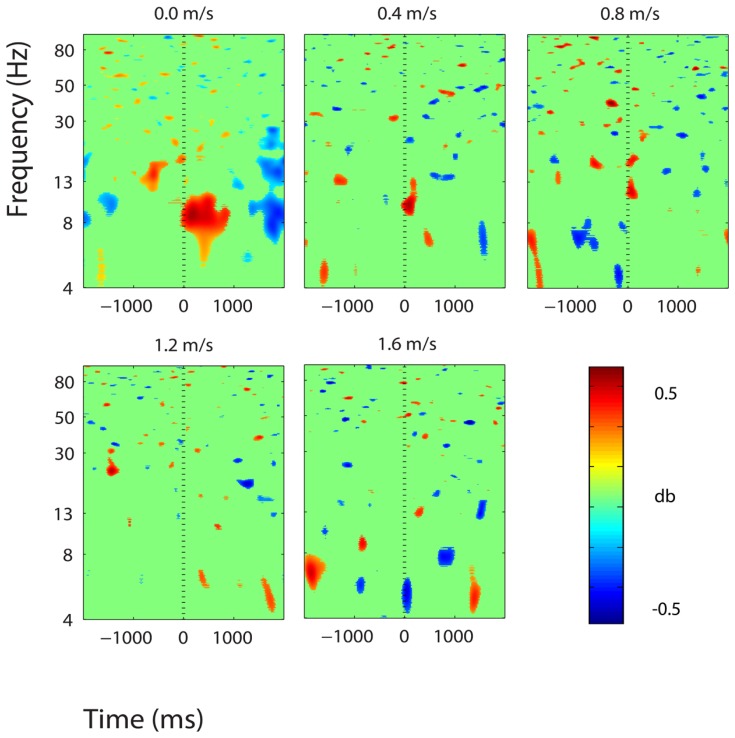
**Event-related spectral perturbation plots showing power change around the presentation of a stimulus (a digit and its position in an imagined grid) in the premotor and supplementary motor cortex (BA 6).** Red represents a power increase from baseline and blue represents a power decrease from baseline. We set non-significant differences to 0 dB (green). The stand condition ERSP is the average of all three standing Brooks spatial working memory task trials.

**FIGURE 9:**
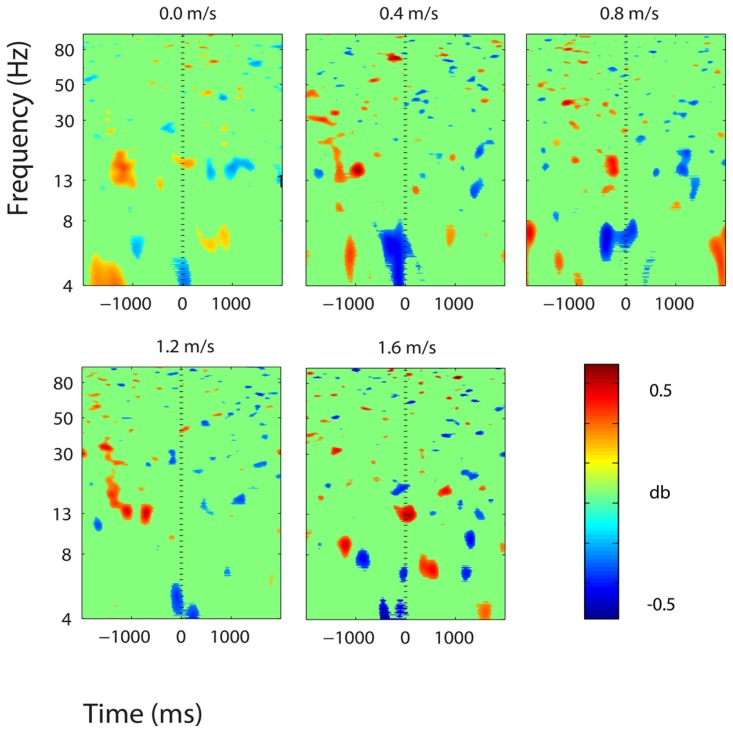
**Event-related spectral perturbation plots showing power change around the presentation of a stimulus (a digit and its position in an imagined grid) in the right superior parietal lobule (BA 5).** Red represents a power increase from baseline and blue represents a power decrease from baseline. We set non-significant differences to 0 dB (green). The stand condition ERSP is the average of all three standing Brooks spatial working memory task trials.

**FIGURE 10:**
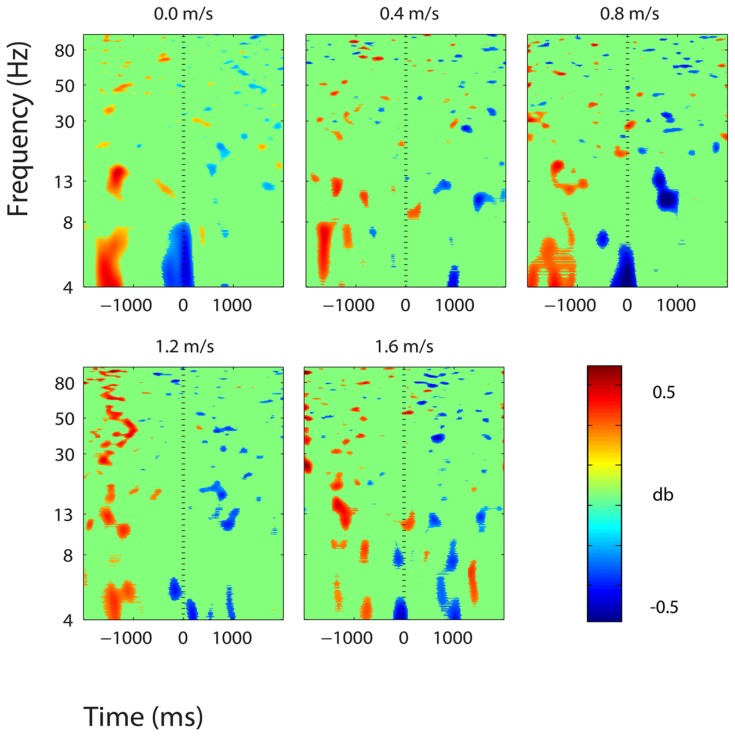
**Event-related spectral perturbation plots showing power change around the presentation of a stimulus (a digit and its position in an imagined grid) in the posterior cingulate cortex (BA 31).** Red represents a power increase from baseline and blue represents a power decrease from baseline. We set non-significant differences to 0 dB (green). The stand condition ERSP is the average of all three standing Brooks spatial working memory task trials.

There were some minor significant spectral shifts in three other clusters, located in the premotor and supplementary motor area, the superior parietal lobule, and the posterior cingulate cortex. In the premotor and supplementary motor area, power in the lower alpha band increased significantly following stimulus presentation during standing, but not during walking (**Figure 8**). In the superior parietal lobule and the posterior cingulate cortex, theta (4–7 Hz) power decreased significantly around stimulus presentation during standing and walking at all speeds (**Figures 9** and **10**).

**FIGURE 11:**
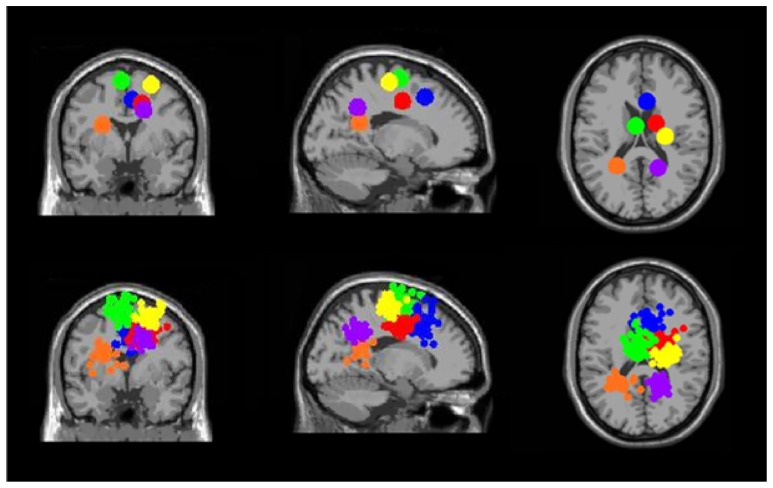
**Clusters containing electrocortical sources from >10 subjects but without significant spectral shifts time-locked to stimulus presentation during the Brooks spatial working memory task.** Green is left premotor and supplementary motor cortex (BA 6), yellow is right premotor and supplementary motor cortex (BA 6), blue is right anterior cingulate (BA 32), red is right anterior cingulate (BA 24), orange is left posterior cingulate (BA 31), and purple is right posterior cingulate (BA 31). From left to right, the top three images show the centroid locations for each cluster from a horizontal, coronal, and sagittal view. The bottom three images show the independent component dipoles for each cluster from the same three perspectives.

**FIGURE 12:**
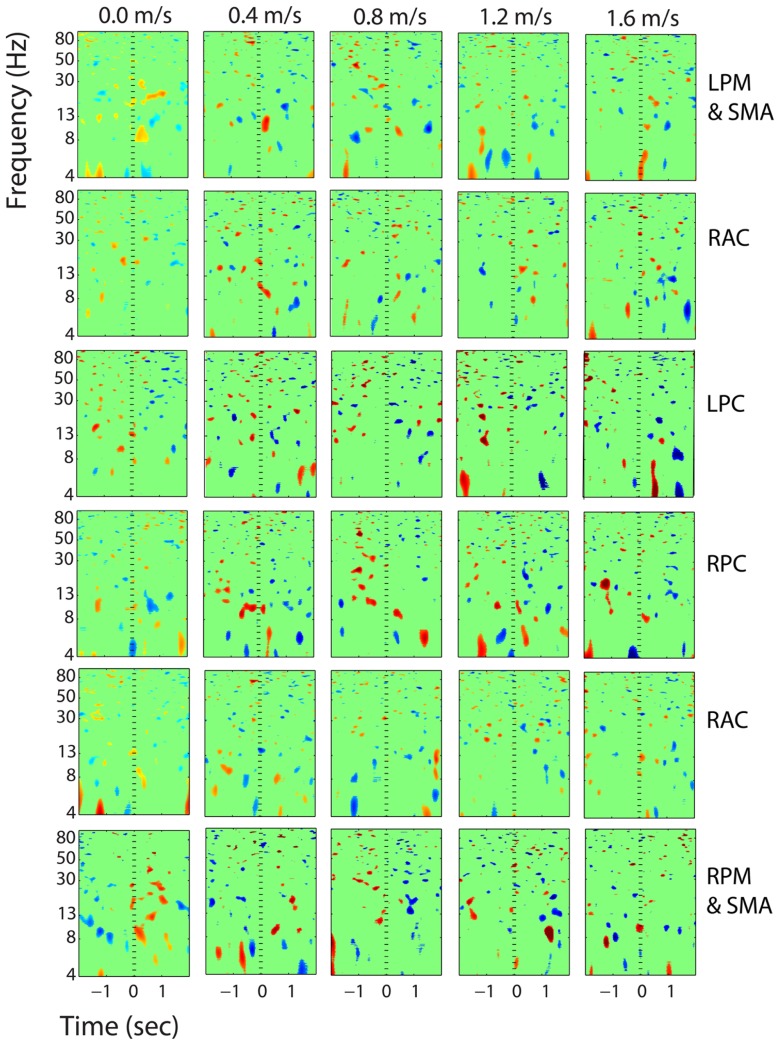
**Event-related spectral perturbation plots showing power change around the presentation of a stimulus (a digit and its position in an imagined grid) in the additional six clusters with independent components from >10 subjects.** Each row is one cortical area. From top to bottom, the cortical areas are left premotor and supplementary motor area (LPM and SMA), right anterior cingulate (RAC), left posterior cingulate (LPC), right posterior cingulate (RPC), and right premotor and supplementary motor area (RPM and SMA). The color scale is from -0.5 to 0.5 db.

Clusters containing independent components from more than half of the subjects (>10) were also located in left and right premotor and supplementary motor area (BA 6), left and right posterior cingulate cortex (BA 31), and right anterior cingulate cortex (BA 24, 32) (**Figure 11**). These six clusters did not have large event-related spectral power changes linked to stimulus presentation that were consistent across walking speeds (**Figure 12**).

For all six clusters showing significant spectral power shifts temporally linked to stimulus presentation, there was no significant effect of walking speed on spectral power. After analysis with nlmefit and the false discovery rate algorithm, for all six clusters, the *p*-value calculated by the false discovery rate algorithm was equal to zero. This means that there were no significant *p*-values, and therefore no significant power changes by speed.

## DISCUSSION

Our results indicate that walking speed does not affect spatial working memory task performance or task-related electrocortical activity in young, healthy subjects within the range of speeds measured here. Our subjects showed no change in response accuracy or reaction time for the Brooks spatial working memory task across walking speeds. Their success rate for the task was about 50% regardless of whether they were standing, walking slowly, or walking quickly. We also found no evidence of change in the electrocortical activity devoted to the cognitive task across walking speeds. The ERSP- graphs in **Figures 6**–**11** reveal similar patterns for standing and all walking speeds (with the exception of the premotor and supplementary motor cortex, **Figure 9**). The mixed effects model showed no significant trend in spectral power change across speeds. If we had found a speed effect in either task performance or electrocortical activity without the other having a speed effect, our results would have been harder to interpret.

Previous studies have shown that performance on some types of cognitive tasks is affected by walking speed. Tomporowski and Audiffren (2013) showed that in older adults performing an executive processing task, response times decreased and response errors increased when walking at faster speeds compared with walking at slower speeds. When walking freely, young and older adults tend to decrease their walking speed when given cognitive tasks to perform during locomotion (Beauchet et al., 2002; Patel et al., 2014). This decrease in motor performance with dual-task walking and cognition suggests that there are common mental resources devoted to both tasks. Thus, we expected to see a change in cognitive performance and electrocortical activity as walking speed increased. However, the majority of studies reporting dual-task performance costs during locomotion have examined elderly or impaired populations. It may be that young, healthy subjects have an increased ability to walk at high speeds and perform a challenging cognitive task with no performance decrease compared with older subjects. In addition, the type of cognitive task may alter the relative amount of dual-task performance cost, as different cognitive tasks rely on different cortical substrates (Smith et al., 1996). When young, healthy subjects have demonstrated a dual-task performance cost (Melzer and Oddsson, 2004; Grabiner and Troy, 2005; Al-Yahya et al., 2009; Verrel et al., 2009; Szturm et al., 2013; Patel et al., 2014), the cognitive task was not a spatial working memory task.

Across all speeds, the addition of the spatial working memory task caused our subjects to step more widely compared to walking without a cognitive task. Wider steps have been associated with more stable gait (Bauby and Kuo, 2000; McAndrew Young and Dingwell, 2012). Past studies on young, healthy subjects performing cognitive tasks during walking have been inconsistent in finding step width changes (Grabiner and Troy, 2005; Siu et al., 2008; Al-Yahya et al., 2009). Our spatial working memory task’s significant effect on step width in young, healthy subjects could be related to its high difficulty level. Subjects correctly placed only about half of the digits at each speed, indicating that the task was very challenging. Patel et al. (2014) found that the complexity of the cognitive task influences the relative dual-task cost during gait. The lack of dual-task effects on gait kinematics for young, healthy subjects in past studies may be related to this difficulty/complexity effect or to the type of cognitive task, as we discussed above.

When we broke down the kinematic results into periods of encoding vs. retrieval for the cognitive task, we found that the retrieval period showed changes in gait indicative of decreased stability. Step width and step width variability were both greater in the phase of the cognitive task that required the subjects to push buttons on the hand-held device (retrieval) compared with the memorization period (encoding). The subjects also took shorter steps during the retrieval period compared to the encoding period. All of these changes in gait kinematics are traditionally seen by individuals that are older and have reduced gait stability (Dubost et al., 2006; Siu et al., 2008; Yogev-Seligmann et al., 2008; Granacher et al., 2011). The encoding task is not unlike many everyday walking tasks such as walking and texting, or walking and dialing a phone number. This suggests that in the real world, performing dual manual and locomotor tasks likely results in humans responding to stability challenges as well.

We found consistent electrocortical activity around stimulus encoding at all walking speeds in the somatosensory association cortex. ERSPs showed substantial alpha (8–13 Hz) synchronization preceding stimulus presentation and alpha desynchronization following stimulus presentation during the Brooks task. This area of the parietal lobe is involved in locating objects in space and is engaged during spatial working memory tasks (Carlesimo et al., 2001). We presented a stimulus every 4 s during the Brooks task. Alpha power in the left, right, and central portions of the somatosensory association cortex increased approximately 1 s before the presentation of the stimulus (a digit and its corresponding position in the grid) and remained elevated until shortly after stimulus presentation. Alpha power in the somatosensory association cortex decreased approximately 0.5 s after stimulus presentation. This could represent neural encoding of the digit and its position in working memory. The alpha power fluctuation was stronger in the right than in the left somatosensory association cortex, which may indicate that this brain area shows a right hemisphere dominance for locating objects in space. Eighteen of the twenty subjects were right-hand dominant.

We found small, significant theta power decreases around stimulus encoding in two brain areas: the right superior parietal lobule and the central posterior cingulate. Theta power modulations during memory tasks have been associated with memory encoding and maintenance by previous electrophysiology studies (Bastiaansen et al., 2002; Friese et al., 2012; Itthipuripat et al., 2013; Lenartowicz et al., 2014). Bastiaansen et al. (2002) found sustained theta power decreases in frontal electrodes during the retention phase of a spatial working memory task. Burke et al. (2013) also found decreases in theta phase synchrony during memory encoding. Many studies have shown theta increases in frontal brain regions during memory encoding and maintenance (Itthipuripat et al., 2013; Lenartowicz et al., 2014). However, we did not find any theta power increases in the frontal brain areas during performance of our task. It could be that during walking, these areas are engaged in gait-related processing, so cognitive task-related electrocortical shifts are undetectable within the gait related electrocortical activity. A host of functional near-infrared spectroscopy studies have found that the frontal cortical areas are highly engaged during human walking, even without a secondary task (Miyai et al., 2001; Suzuki et al., 2004; Harada et al., 2009). In addition, in a previous study from our lab, we also found electrocortical evidence of frontal cortical involvement in human walking without a cognitive task (Gwin et al., 2011). Our analysis of spectral perturbations in electrocortical activity was specifically limited to changes synchronized to stimulus presentation.

On a similar note, activity in the premotor and supplementary motor cortex may demonstrate some overlap between cognitive and motor processing (**Figure 9**). During standing, power in the lower alpha band increased around stimulus presentation, and alpha and beta power decreased shortly thereafter. During walking, this effect was not visible. Studies have shown that the premotor and supplementary motor cortex is engaged during walking (Gwin et al., 2011). During walking this area was likely engaged in motor processing, and cognitive-related activity was either not present or undetectable.

Some researchers have questioned how much mechanical artifact is present in walking EEG data synchronized to the gait cycle (Castermans et al., 2014). Previous studies have shown that EEG during walking is viable for non-gait synchronized data, as the approach of synchronizing EEG data to cognitive events not coupled to gait allows mechanical artifacts to wash out (Gramann et al., 2010). We observed the same pattern of neural activity at all speeds from standing (0.0 m/s) to very fast walking (1.6 m/s). This result indicates that EEG and ICA can reveal neural activity from a continuous cognitive task even when a subject is moving quickly. To our knowledge, our results are the first to show that high-density EEG and ICA can allow for the study of continuous cognitive dynamics at high walking speeds. This has important implications for the field of mobile brain imaging, as it suggests EEG could be used even in real-world environments that include walking at normal speeds.

Our study had some limitations that prevent us from drawing broad conclusions about the effects of speed on spatial working memory. First, we only examined young, healthy subjects. It is possible that we would have seen a larger effect in gait parameters and task performance if we had tested elderly subjects. Future studies should examine the neural and performance responses of elderly and neurologically impaired individuals. Second, we only examined a single cognitive task and a single cognitive task difficulty level. While unlikely, it is possible that an easier task or a harder task may have resulted in a different outcome. Third, the majority of the subjects in this study were males. There is some evidence that males may walk with increased variability under dual-task conditions. Hollman et al. (2011) had older adults walk and perform a backward spelling task. Both genders increased their variability when walking with the cognitive task, however, the variability increase during dual-task walking was greater in men than women. Finally, we did not assess our subjects’ preferred speed. A study by Schaefer et al. (2010) showed that children and young adults improved their cognitive performance on a working memory task while walking at their preferred speed on a treadmill. The ideal dual-task cost study would include a range of cognitive tasks and difficulty levels, to separate out overlap in neural substrates between walking and various cognitive tasks.

Our findings raise some interesting questions about dual-tasking. A popular view suggests that because walking requires mental effort, performing both walking and thinking can decrease thinking ability compared with thinking while standing still (Kahneman, 2011). Our results do not support that conclusion. For our spatial working memory task, mental performance did not change for walking vs. standing. Similarly, a recent study on the use of a treadmill desk found that human subjects performed equally well on a range of cognitive tasks for slow walking compared to standing (Alderman et al., 2013). John et al. (2009) also reported that subjects performing slow walking at a treadmill desk had no significant differences in selective attention, processing speed, or reading comprehension compared to sitting at a desk. Our study only examined a single cognitive task, but we found no detrimental effects over a wide range of walking speeds on cognitive ability.

Other researchers have suggested that moving your body may actually improve creativity. Slepian and Ambady (2012) found that subjects performed better on cognitive tasks related to creativity when moving their arms in fluid movements compared with not moving their arms. Longer term studies have found that chronic exercise also seems to increase creativity, but that is on the time scale of weeks or months (Flaherty, 2011). Putting together all of these observations with our own results, we suggest that people should not be concerned that walking while thinking will impair their cognitive performance. All in all, our study demonstrates that young, healthy subjects perform equally well on a challenging spatial working memory task during walking at a range of speeds as during standing. Given the many benefits of walking (Rippe et al., 1988; Hardman and Morris, 1998), people should be encouraged to walk and think whenever possible.

## AUTHOR CONTRIBUTIONS

Daniel P. Ferris and Julia E. Kline designed the experiment. Julia E. Kline and Katherine Poggensee carried out all experimental procedures. Julia E. Kline performed the data analysis. Katherine Poggensee performed the majority of the statistical analysis. Daniel P. Ferris, Julia E. Kline, and Katherine Poggensee wrote and edited the manuscript.

## Conflict of Interest Statement

The authors declare that the research was conducted in the absence of any commercial or financial relationships that could be construed as a potential conflict of interest.
